# The choice of corporate social responsibility strategies under rivalry: Whether to increase socially responsible product characteristics or enhance relationship with buyers

**DOI:** 10.1371/journal.pone.0343679

**Published:** 2026-03-04

**Authors:** Xiaoyang Zhao

**Affiliations:** School of Public Policy and Management, Anhui Jianzhu University, Hefei, Anhui, China; University of Naples Federico II: Universita degli Studi di Napoli Federico II, ITALY

## Abstract

Corporate social responsibility (CSR) theory emphasizes both CSR characteristics in products and CSR activities to enhance relationships with buyers. However, there are theoretical gaps regarding the factors that influence firms’ CSR strategy choices in a competitive setting. This study develops a biform game model to study the trade-off between product-oriented strategies and relationship-oriented CSR strategies. The model demonstrates that factors such as information asymmetry, product value, market segment size, transaction costs, bargaining power, and other market conditions significantly influence firms’ CSR strategies. It identifies conditions under which strategic heterogeneity, strategic homogeneity, and a parameter space with multiple strategic heterogeneity equilibria can exist. When the value derived from product- and relationship-oriented strategies is significantly high, firms tend to adopt these respective strategies. In a low information asymmetry context, firms with lower bargaining power are more likely to choose a relationship-oriented strategy, whereas in a high information asymmetry context, these firms are inclined to adopt a product-oriented strategy. Regardless of the level of information asymmetry, firms with larger market scales tend to favor a product-oriented strategy. Additionally, the model integrates the synergy between business strategy and CSR strategy, which further shapes the trade-offs firms encounter in their CSR strategy choices. This study offers new insights into CSR strategy choices and the resulting market outcomes in competitive environments.

## Introduction

Socially irresponsible activities, such as false marketing, food safety violations, environmental pollution, financial fraud, and more recently, data privacy infringements [1,2], have underscored the need for social responsibilities to be internalized into the management and governance of enterprises [3–5]. Scholars and practitioners have increasingly recognized that corporate social responsibility (CSR) is an important strategic aspect with significant implications for firms’ performance [6,7]. Consequently, the strategic choices regarding which CSR domains a firm should focus on have become a prominent topic on research agendas, rooted in the concept of ‘the business case for CSR’ [1,8,9].

From a value-based perspective, CSR strategy choices involve mechanisms that determine how CSR activities can create and capture value [10,11]. Fundamentally, a firm can leverage strategic CSR activities to achieve higher performance either by enhancing product-related CSR characteristics to increase value creation or by strengthening customer relationships to foster trust and reduce transaction costs [1]. In this context, product-oriented CSR strategies encompass process and product innovations that improve the characteristics of safety, health, and environmental protection, with recent practice emphasizing customer needs to upgrade user CSR experiences. A representative example is Honor’s product-centric CSR approach. The company integrates social responsibility directly into its core product offerings through technological innovation, such as its Oasis Eye Protection Screen technology [12]. By investing in R&D to improve screen safety and reduce eye strain for hundreds of millions of users, Honor embeds public health value into the functionality of its smartphones and tablets, demonstrating how CSR can be driven through product attribute enhancement. Relationship-oriented CSR strategies may involve the philanthropy and donations that build reputation and trust with a focal firm’s customers [13–15]. For instance, in the Chinese dairy industry, Yili Group adopts a distinctively relationship-oriented approach by focusing on building deep social connections beyond its immediate consumer transactions. Its initiatives, such as the “Yili Nutrition 2020” poverty alleviation project and a recent billion-RMB fertility subsidy plan, are designed to strengthen community bonds and address broad societal challenges [16]. This strategy prioritizes long-term trust and goodwill through social engagement rather than direct product enhancement, aiming to foster a supportive societal environment and solidify its license to operate.

However, the CSR literature has primarily focused on empirical evidence of average CSR performance implications [17,18], and thus falls short in explaining the micro-level mechanisms behind the choices of product-oriented and relationship-oriented CSR strategies, particularly when considering both value creation and value capture micro-foundations in a competitive setting [19]. To date, scholars in strategic management have explored the extent to which CSR contributes to firm performance [20,21]. Both positive and negative effects have been observed in the relationship between CSR and firm performance [22]. The debate continues due to limitations in empirical methods that hinder the differentiation among CSR choices [23]. Exploring firms’ CSR choices in competitive settings may help resolve this debate and contribute to CSR strategy literature. Currently, there is a paucity of theoretical research on the strategic aspects of different CSR activities, particularly regarding how CSR helps firms create and capture value in a competitive environment.

This paper explores the micro-foundations of CSR strategy choices using the framework of biform game theory, which formally decomposes the performance implications of firms’ CSR strategies into two related challenges: value creation and value capture [24–26]. By doing so, this paper provides new insights into the theory of CSR strategy choices under competitive rivalry. This study builds a formal biform game model to include product- and relationship-oriented CSR strategies that shape the competitive landscape and determine market outcome. Through the analysis of the biform game model, it provides a more granular understanding of CSR strategy choices. This paper extends the strategic CSR literature that focuses on performance implications of CSR strategies by revealing the micro-foundation underlying firms’ CSR strategy choices in a competitive market setting.

## Literature review

Corporate social responsibility strategies have increasingly become integral to firms’ competitive positioning, particularly in environments marked by intense rivalry [18,27]. Research indicates that companies often adopt CSR initiatives not only for ethical reasons but also to differentiate themselves from competitors, thereby gaining a strategic edge [28,29]. For instance, studies show that CSR can enhance firm performance by fostering stakeholder trust and improving market reputation, which is crucial under competitive pressures [30]. Furthermore, in highly competitive markets, firms may mimic rivals’ CSR activities to neutralize potential disadvantages, suggesting that rivalry influences the convergence of CSR strategies across industries [1]. The dynamic competition underscores the role of CSR as a tool for maintaining parity or achieving superiority in rivalrous settings [24].

The literature on CSR strategies has revealed strategic typologies distinguishing between product-oriented and relationship-oriented approaches [1]. Product-oriented CSR strategies focus on integrating social and environmental value into a firm’s products through innovations that enhance product safety, health, and environmental attributes. This approach aligned with the resource-based view, where such investments create unique, difficult-to-imitate resources that drive differentiation and consumer value [10]. In contrast, relationship-oriented CSR strategies emphasize building trust and long-term bonds with stakeholders through philanthropy, community engagement, and strategic donations, thereby strengthening reputation and social legitimacy, as explained by stakeholder theory [15,31]. This theoretical divide reflects the strategic choice firms face between enhancing product-specific characteristics and cultivating relational capital underscoring CSR’s role in achieving competitive advantage when aligned with core business objective [9,32].

When choosing between enhancing socially responsible product characteristics or strengthening buyer relationships, firms must consider how these strategies interact with competitive forces [33]. Literature highlights that product-focused CSR, such as incorporating sustainable features, can appeal to environmentally conscious consumers and create differentiation, but it requires significant investment and may be easily imitated by rivals [23,34]. In contrast, relationship-oriented CSR strategies, like building long-term partnerships with buyers through ethical sourcing or community engagement, can foster loyalty and reduce switching costs, providing a more defensible competitive advantage in oligopolistic markets [19]. Ultimately, the efficacy of these approaches depends on the intensity of rivalry and stakeholder expectations, as evidenced by frameworks that link CSR to value chain integration and societal impact [1].

## The model

This study begins by setting up a baseline biform game model which specifies micro-foundation of agents’ interactive activities and outcomes. The rationale for employing a biform game, rather than a standard non-cooperative approach such as Nash equilibrium or Stackelberg competition, lies in its ability to capture both the strategic moves that shape the competitive environment and the subsequent cooperative bargaining over value distribution [25]. While classical non-cooperative models focus predominantly on equilibrium outcomes under competition, they often overlook how ex post cooperation and bargaining power influence value capture—particularly in settings where players can form coalitions or adjust alliances endogenously [35]. The biform framework explicitly integrates a non-cooperative stage with a cooperative stage (e.g., value division via solutions such as the core or Shapley value), thereby allowing analysts to examine how initial strategic choices affect not only total value creation but also its distribution among stakeholders. This dual structure is especially suitable for modeling contexts where firms first compete to establish strategic positions and then cooperate to realize joint gains in the study.

In the model, there are two groups of players whose interactions influence their possible coalition structure for value creation and value capture. The model is divided into two stages including a competitive stage and a cooperative stage [25,36]. In the competitive stage, players choose their CSR strategies under rivalry which leads to different competitive landscapes [37]. Based on the competitive landscapes, the possibilities of player coalitions and value creation can emerge during the cooperative stage. Depending on the potential cooperative structures and the distribution of bargaining power, a player’s added value, minimum guaranteed value, and value capture from the cooperative stage can be analyzed using the concept of the “core.” The “core” represents the value a player appropriates from the network, highlighting the player’s share of the cooperative gains [35].

The concept of the “core” is fundamental in biform game theory, representing the outcomes of both competitive and cooperative interactions [25]. It defines the value distribution among players within a coalitional network, considering the constraints of a focal player’s added value and its incentive to improve its position by potentially breaking away from an existing coalition [24,38]. Value capture by players is determined by the potential coalitional structures of value creation, provided that feasibility and consistency are maintained. Feasibility requires that the total value created matches the total value captured, while consistency ensures that no subgroup is motivated to leave a coalition if they can create and capture more value independently [25]. The “core” encompasses all possible value distributions that satisfy these two conditions, reflecting the outcomes of CSR strategic choices during competitive interactions. Therefore, the “core” is a key concept as the outcome of a biform game model. Specifically, the “core” illustrates that each agent could capture value within an interval ${[\pi }^{min},\;\pi^{max}]$. The point estimate for an agent’s value capture is $\pi^{min}+\alpha (\pi^{max}-\pi^{min})$, where α denotes the bargaining power of a focal agent. The “core”, rather than the Shapley value or kernel, is used in the model because it offers deeper insights into agents’ strategic choices by incorporating factors such as industrial structure, competitive advantage, and bargaining power.

In the model, two firms compete for buyers in a segmented market. During the competitive stage, each firm chooses between two types of CSR strategies: one that focuses on creating value through enhanced socially responsible product characteristics, such as safety, health, and environmental protection, and another that aims to strengthen relationships with buyers, thereby increasing bargaining power and reducing transaction costs. The product-oriented CSR strategy enhances the value creation that a firm can capture in the cooperative stage. In contrast, the relationship-oriented CSR strategy lowers transaction costs and boosts bargaining power, leading to a broader range of value creation and a larger share of captured value within this new range [37]. Depending on the landscape shaped by the firms’ CSR strategy choices in the first stage, the two firms compete for buyers across three market segments and ultimately determine their value capture in the cooperative stage. Table 1 below illustrates the process of these two stages and the key parameters in the model, highlighting the main activities and interactions in both the competitive and cooperative stages.

**Table 1:** Stages and key parameters of the model.

Initial conditions	First stage-competitive stage	Second stage-cooperative stage
In initial state, Firms *i* = 1, 2 create value $v_{iX}^{\text{0}},\;v_{iY}^{\text{0}}\text{ and }v_{iZ}^{\text{0}}$ for buyers in three market segments *m* = *X*, *Y*, Z, with their own initial bargaining power $\alpha_{i}^{\text{0}}$.	Each firm *i* chooses a CSR strategy $s_{i}\in \{P,R\}$, where *P* strategy increases value creation by Δv. Inputting a fixed cost γ, *R* strategy decreases its transaction costs by Δt and increases bargaining power by Δα. Solved for pure-strategy Nash equilibrium (PSNE) using backwards induction.	Firms compete for buyers in three market segments. Coalitions are built based on the competitive landscape determined in the first stage. Value capture by agents in our model could be derived based on the cooperative structure. Solved using the “core” from cooperative game theory.

To study the choice between product-oriented and relationship-oriented CSR strategies, a fundamental theoretical assumption is that firms face organizational trade-offs when deciding whether to enhance product characteristics or strengthen relationships with buyers [39]. These trade-offs arise due to limitations in resources, managerial recognition, or attention [28,40,41], which constrain managers’ ability to balance CSR strategies within a specific timeframe. This trade-off between product CSR characteristics and relationships mirrors the tension between explorative and exploitative activities in firms [42]. Such strategic trade-offs are common management challenges, reflecting paradoxes in resource allocation and organizational design [43,44].

### Players and market settings for value creation

This study first defines the set of players in the market for the model, among whom value is created and captured in potential coalitions. In this market, there are two firms, indexed as *i* = 1, 2, competing to provide products to a set of buyers where *n* ≥ 3. Let *N* represent the set of all players, including the two firms and the *n* buyers. Each buyer belongs to one of three market segments, indexed as *m* = *X*, *Y, Z*. Buyers are identical within each segment but differ across segments. Let $n_{m}\geq \text{1}$ denote the number of buyers in segment *m*, so we have $n_{X}+n_{Y}+n_{Z}=n$.

Firms and buyers can only create value within a coalition, meaning that value creation hinges on a transaction between a firm and a buyer. Each buyer has demand for only one unit of product from a single firm. The value creation by firm *i* with a buyer in segment *m* is denoted as $v_{im}\geq \text{0}$. This created value in the coalition reflects the difference between the buyer’s willingness-to-pay for firm *i*’s product and firm *i*’s marginal costs of production plus the transaction costs associated with the firm-buyer interaction. For a rational buyer, the willingness to pay for firm *i*’s product is primarily influenced by the product’s utility value. Transaction costs arise from processes such as information search, product comparison, and forming formal or informal agreements [45]. Consequently, differences in the value created by firm *i* for buyers in segments X, Y, and Z (denote as $v_{iX}$, $v_{iY}$, and $v_{iZ}$) reflect heterogeneity in buyer preferences for product features, as well as variations in production and transaction costs. Based on the value creation in a coalition, firm *i* can capture a share of its added value from the created value $v_{im}$ according to firm *i*’s initial bargaining power $\alpha_{im}=\alpha_{im}^{\text{0}}\in$ (0,1). Since $v_{im}$ is a constant value within a specific market segment, it is assumed that firms do not face capacity constraints in producing additional products. Additionally, there are no economies of scale influencing the reduction of marginal costs.

Much of the theorizing on value-oriented CSR within strategy and economics does not differentiate between home markets and mutable markets. A key distinction in the model is that CSR activities can alter the market transaction structure. Typically, buyers in the mutable market may be influenced by CSR activities and shift to a firm’s home market. In contrast, CSR activities generally do not have a significant impact on the coalition structures of firms and buyers within their home market. In the model, parameters are set to capture this distinction, with value creation in a firm’s home market being greater than in other market segments. Neither firm holds a competitive advantage in the mutable market:


$$v_{\text{1}X}^{\text{0}}\;=v_{h},\;v_{\text{1}Z}^{\text{0}}=\;v_{l},\;v_{\text{2}X}^{\text{0}}=v_{l},\;v_{\text{2}Z}^{\text{0}}=v_{h},\;v_{\text{1}Y}^{\text{0}}\;=v_{\text{2}Y}^{\text{0}}=v_{m}$$ (1)

The parameters of value creation above indicates that Firm 1 creates more value in its home segment *X*, where Firm 1’s added value for each buyer in *X* is ${AV}_{\text{1}X}=v_{h}\;-v_{l}\;>\text{0}$; Firm 2 creates more value in its home segment *Z*, where Firm 2’s added value for each buyer in *Z* is ${AV}_{\text{2}Z}=v_{h}\;-v_{l}>\text{0}$. In market segment *Y*, Firm 1 and 2 provide the same value. Firms’ added value is ${AV}_{\text{1}Z}={AV}_{\text{2}Z}=\text{0}$. The zero added value means that a firm has added value only in its home segment in the initial condition. All value created by firms and buyers in market *Y* is appropriated by buyers since firms compete for serving these buyers and no one has a competitive advantage.

### Firms’ CSR strategic choices under rivalry in the competitive stage

#### The product-oriented CSR strategy.

The product-oriented CSR strategy $s_{i}=P$ aims to enhance the value creation for buyers by introducing socially responsible product characteristics. For firm *i* adopting the *P* strategy, the value creation for buyers in a market segment increases to $v_{im}^{\prime }=v_{im}^{\text{0}}+\Delta v_{im}$, where $\Delta v_{im}$≥0 represents the additional increase in buyers’ willingness to pay for the product. This enhancement comes at the cost of a higher marginal production cost. Under the theoretical assumption that CSR features in a product are independent of the product’s utility value and are equally valued by all buyers, the study sets $\Delta v_{im}=\Delta v$, indicating a constant increase in value creation across segmented markets for simplicity. The product-oriented strategy considers CSR characteristics that enhance value creation. This includes the use of socially responsible raw materials, sustainable production processes, cause-related marketing, and recycling initiatives that improve social benefits and sustainability, thereby increasing value [46]. For example, in the food industry, adopting recycled packaging would be an example of a product-oriented strategy.

#### The relationship-oriented CSR strategy.

The relationship-oriented CSR strategy $s_{i}=R$ seeks to strengthen firm *i*’s relationship with buyers, through activities such as social causes marketing, sponsorship, corporate philanthropy, and corporate volunteering [46]. Many firms invest resources in these initiatives to address social problems and enhance social welfare in both local and global communities [47]. For a firm employing the *R* strategy, transaction costs between the firm and its buyers decrease by Δt. Additionally, the firm’s bargaining power increases to $\alpha_{i}=\alpha_{i}^{\text{0}}+\Delta \alpha \in$ (0,1). This increase in bargaining power is partly because buyers tend to trust firms that engage in relationship-oriented CSR strategies [15] and are often willing to reward socially responsible firms by compromising during negotiations [13,48]. Unlike product-oriented CSR strategies, relationship-oriented strategies often involve a fixed investment γ rather than additional marginal costs. The relationship-oriented CSR strategy includes activities such as philanthropy and donations [46]. However, the *R* strategy does not alter the buyer’s willingness to pay, as it does not enhance the product’s utility value for customers.

Moreover, when both firms adopt the relationship-oriented CSR strategy, the distinctive advantages typically gained from such strategies diminish. This is because the effectiveness of relationship-oriented CSR strategies largely depends on the relative differences in relationships between firms and their customers. When both firms pursue similar relationship strategies, buyers are less likely to differentiate between them during transactions and bargaining. Consequently, the comparative advantage that one firm might gain from improved relationships is neutralized, leading to no changes in transaction costs or bargaining power for either firm.

Further, this study assumes that the value created by both the *P* strategy and *R* strategy is less than the value differences between a firm’s home market and its rival’s home market: $v_{h}\;-v_{l}>\Delta v,\;\Delta t$. This implies that CSR activities alone are insufficient for a firm to penetrate its competitor’s home market. However, these strategies can be effective in capturing value within the mutable market by allowing a firm to dominate that space and capture value from the resulting coalitions.

### Value capture of players in the cooperative stage

Based on the framework of biform game theory, the “core” of a cooperative game among industry participants determines players’ value capture in the model [35,36]. The total value created by all *N* players in the model is denoted by *V(N)*. When efficiency is satisfied, *N* players join a coalition network that creates the maximum possible value. That is, a firm provides products for buyers in its home segment *m.* Then, the total value created by all players in the model is $V(N)=n_{X}max\left\{v_{\text{1}X},v_{\text{2}X}\right\}+n_{Y}max\left\{v_{\text{1}Y},v_{\text{2}Y}\right\}+n_{Z}max\{v_{\text{1}Z},v_{\text{2}Z}\}$. The value captured by firm *i* is denoted by *Π*_*i*_, and the value captured by a buyer in market segment *m* is denoted by *u*_*m*_.

As a standard solution for a player’s value capture in a biform game model, the “core” defines a possible range of value captured by a player. The upper bound of that range for firm *i* in the coalition with a buyer in segment *m* is determined by the firm’s added value ${AV}_{im}\;=\;max\{v_{im}-v_{-im},\;\text{0}\}$. Firm *i*’s total added value is then $n_{X}{AV}_{iX}+n_{Y}{AV}_{iY}+n_{Z}{AV}_{iZ}$. The lower bound of that range is given by a player’s added value for alternative coalitions of players. In the model, since firm *i* is already part of the coalitions that maximize value creation in its home market, the lower bound of its value capture is zero. Within that range, the point estimate of a player’s value capture is then determined by a player’s bargaining power. The model introduces a bargaining power parameter $\alpha_{im}\in (\text{0},\;\text{1})$ that determines how much of the value firm *i* captures within the range of possible value capture [37]. Consequently, firm *i*’s value capture (*Π*_*i*_) and a buyer’s value capture in segment *m* (*u*_*m*_) are as follows:


$${\mathrm{\backslash itPi}}_{i}=\;{n_{X}\alpha }_{iX}{AV}_{iX}+{n_{Y}\alpha }_{iY}{AV}_{iY}+{n_{Z}\alpha }_{iZ}{AV}_{iZ}$$ (2)

$$u_{m}=max\{v_{\text{1}m},v_{\text{2}m}\}-\alpha_{\text{1}X}{AV}_{\text{1}X}-\alpha_{\text{2}X}{AV}_{\text{2}X}$$ (3)

Then, according to the efficiency principle, the total value captured by all buyers is $U=n_{X}u_{X}+n_{Y}u_{Y}+n_{Z}u_{Z}=v(N)-{\mathrm{\backslash itPi}}_{\text{1}}-{\mathrm{\backslash itPi}}_{\text{2}}$.

### Solution for the biform model

As shown in Table 1, firms simultaneously choose their CSR strategies in the first stage. Firm *i* can choose a product-oriented CSR strategy ($s_{i}=P$) or a relationship-oriented strategy ($s_{i}=R$). If firm *i* pursues the *P* strategy, the willingness-to-pay level increases by Δv, while the bargaining power remains at $\alpha_{im}=\alpha_{im}^{\text{0}}$. Conversely, if firm *i* pursues the *R* strategy, its bargaining power increases to $\alpha_{i}^{\prime }=\alpha_{i}^{\text{0}}\;+\Delta \alpha$, and transaction costs between the firm and buyers decrease by Δt. The value distribution in the cooperative stage is then shaped by the CSR strategies chosen by the firms.

This paper analyzes the model using backward induction. First, it derives the firms’ value capture in the cooperative stage by applying the “core”, taking into account all possible combinations of CSR strategy choices made by the firms during the competitive stage. Then, it solves for the pure-strategy Nash equilibria (PSNE) in the first stage where firm *i* chooses a pure strategy $s_{i}$ to maximize its expected value capture in the second stage E(${\mathrm{\backslash itPi}}_{i}|s_{i},s_{-i}$), given the strategy choice of its rival $s_{-i}$. For simplicity and tractability, the model does not consider mixed-strategy equilibria in this analysis.

## The Equilibrium: Product- or relationship- oriented CSR strategies?

In this section, this study analyzes when firms prefer product-oriented versus relationship-oriented CSR strategies and what determines the equilibrium of CSR strategies under rivalry. It uses backward induction to analyze the equilibrium of the model. Given the importance of information on the effectiveness of CSR strategies, it addresses these questions by examining the equilibrium of firms’ CSR strategy choices in both low and high information asymmetry contexts [49]. In a low information asymmetry context, buyers can accurately perceive product value, and transaction costs are relatively low. Here, the added value from a product-oriented CSR strategy outweighs the reduction in transaction costs, meaning Δv−Δt>0. Conversely, in a high information asymmetry context, buyers have difficulty perceiving product value, and transaction costs are relatively high. In this scenario, the added value from a product-oriented CSR strategy is less likely to outweigh the reduced transaction costs, indicating that Δv−Δt<0.

### Low information asymmetry industrial context

The condition for firms’ CSR strategy portfolio ($s_{i}^{*},s_{-i}^{*}$) to be a pure-strategy Nash equilibrium (PSNE) is that neither firm has an incentive to unilaterally deviate to another strategy. Thus, for an equilibrium, the following must holds for *i* = 1, 2: *E*(${\mathrm{\backslash itPi}}_{i}{|s}_{i}^{*},s_{-i}^{*}$) ≥ *E*$\left({\mathrm{\backslash itPi}}_{i}|\hat{s_{i}^{*}},s_{-i}^{*}\right)$ for any $\hat{s_{i}^{*}}\neq s_{i}^{*}$. Based on the characteristic functions, this study derives the “core” of value appropriation and the point estimates consistent with the “core” for the firms. In an industrial setting with low information asymmetry where Δv−Δt>0, the point estimates of the baseline model are listed in Table 2.

**Table 2:** The payoff matrix of the game between firms in a low information asymmetry context.

Firm 1	Firm 2	
	*P*	*R*
*P*	${n_{X}\alpha }_{\text{1}}(v_{h}\;-v_{l})$, ${n_{Z}\alpha }_{\text{2}}(v_{h}\;-v_{l})$	${n_{X}\alpha }_{\text{1}}\left(v_{h}\;-v_{l}+\Delta v-\Delta t\right)+{n_{Y}\alpha }_{\text{1}}(\Delta v-\Delta t)$, $\begin{array}{c} {n_{Z}(\alpha }_{\text{2}}+\Delta \alpha )\left(v_{h}\;-v_{l}-\Delta v+\Delta t\right)-\gamma \end{array}$
*R*	$\begin{array}{c} {n_{X}(\alpha }_{\text{1}}+\Delta \alpha )\left(v_{h}\;-v_{l}-\Delta v+\Delta t\right)-\gamma \end{array}$, ${n_{Z}\alpha }_{\text{2}}\left(v_{h}\;-v_{l}+\Delta v-\Delta t\right)+{n_{Y}\alpha }_{\text{2}}(\Delta v-\Delta t)$	${n_{X}\alpha }_{\text{1}}\left(v_{h}\;-v_{l}\right)-\gamma$, ${n_{Z}\alpha }_{\text{2}}(v_{h}\;-v_{l})-\gamma$

Based on the payoff matrix, the equilibrium of firms’ CSR strategies is summarized in Proposition 1.

Proposition 1. *Depending on parameters, in a low information asymmetry setting, there is a symmetric equilibrium where both firms choose the product-oriented CSR strategy (P, P), and two possible asymmetric equilibria where one firm chooses a product-oriented CSR strategy, and the other firm chooses a relationship-oriented CSR strategy, (P, R) or (R, P). Formally*:

(i) *There exists a threshold* $\Delta {\overset{―}{\left(v-t\right)}}_{\left(P,P\right)}=max\{\frac{\Delta \alpha \left(v_{h}\;-v_{l}\right)-\frac{\gamma }{n_{X}},\;}{\alpha_{\text{1}}+\Delta \alpha },\frac{\Delta \alpha \left(v_{h}\;-v_{l}\right)-\frac{\gamma }{n_{Z}},\;}{\alpha_{\text{2}}+\Delta \alpha }\}\geq \text{0}$ *such that (P, P) is an equilibrium if and only if* $\Delta (v-t)\geq \Delta ({\overset{―}{v-t})}_{\left(P,P\right)}$;
(ii) *Either (P, R) or (R, P) is the unique equilibrium if and only if* $min\{\frac{\Delta \alpha \left(v_{h}\;-v_{l}\right)-\frac{\gamma }{n_{X}},\;}{\alpha_{\text{1}}+\Delta \alpha },\frac{\Delta \alpha \left(v_{h}\;-v_{l}\right)-\frac{\gamma }{n_{Z}},\;}{\alpha_{\text{2}}+\Delta \alpha }\}\leq \Delta (v-t)\leq max\{\frac{\Delta \alpha \left(v_{h}\;-v_{l}\right)-\frac{\gamma }{n_{X}},\;}{\alpha_{\text{1}}+\Delta \alpha },\frac{\Delta \alpha \left(v_{h}\;-v_{l}\right)-\frac{\gamma }{n_{Z}},\;}{\alpha_{\text{2}}+\Delta \alpha }\}$.
(iii) *Either (P, R) or (R, P) is a possible equilibrium if* $\Delta (v-t)\leq min\{\frac{\Delta \alpha \left(v_{h}\;-v_{l}\right)-\frac{\gamma }{n_{X}},\;}{\alpha_{\text{1}}+\Delta \alpha },\frac{\Delta \alpha \left(v_{h}\;-v_{l}\right)-\frac{\gamma }{n_{Z}},\;}{\alpha_{\text{2}}+\Delta \alpha }\}$.

Result (*i*) indicates that both firms will adopt a product-oriented CSR strategy when the returns from the *P* strategy are sufficiently high. For example, the (*P*, *P*) equilibrium can be observed in industries like the dairy industry, where firms use environmentally friendly materials for packaging. In this scenario, product CSR characteristics become a key competitive approach for rival firms. In contrast, the equilibrium where both firms choose a relationship-oriented CSR strategy (*R*, *R*) does not hold in a low information asymmetry context. This is because the benefits of enhanced bargaining power and reduced transaction costs from the R strategy are offset by competitors’ use of the R strategy. As a result, firms cannot increase their profits in their home markets nor penetrate the mutable market segment.

In instances where $min\{\frac{\Delta \alpha \left(v_{h}\;-v_{l}\right)-\frac{\gamma }{n_{X}},\;}{\alpha_{\text{1}}+\Delta \alpha },\frac{\Delta \alpha \left(v_{h}\;-v_{l}\right)-\frac{\gamma }{n_{Z}},\;}{\alpha_{\text{2}}+\Delta \alpha }\}\leq \Delta (v-t)\leq max\{\frac{\Delta \alpha \left(v_{h}\;-v_{l}\right)-\frac{\gamma }{n_{X}},\;}{\alpha_{\text{1}}+\Delta \alpha },\frac{\Delta \alpha \left(v_{h}\;-v_{l}\right)-\frac{\gamma }{n_{Z}},\;}{\alpha_{\text{2}}+\Delta \alpha }\}$, a unique asymmetric equilibrium arises, where the firms choose (*P, R*) or (*R, P*). The outcome of this strategic asymmetry is mainly influenced by the size of the firms’ home markets and their initial bargaining power. When $\frac{\Delta \alpha \left(v_{h}\;-v_{l}\right)-\frac{\gamma }{n_{X}},\;}{\alpha_{\text{1}}+\Delta \alpha }\geq \frac{\Delta \alpha \left(v_{h}\;-v_{l}\right)-\frac{\gamma }{n_{Z}},\;}{\alpha_{\text{2}}+\Delta \alpha }$, (*R, P*) is the unique equilibrium. This means that Firm 1 will choose the *R* strategy while Firm 2 opts for the *P* strategy. When $\frac{\Delta \alpha \left(v_{h}\;-v_{l}\right)-\frac{\gamma }{n_{X}},\;}{\alpha_{\text{1}}+\Delta \alpha }\leq \frac{\Delta \alpha \left(v_{h}\;-v_{l}\right)-\frac{\gamma }{n_{Z}},\;}{\alpha_{\text{2}}+\Delta \alpha }$, (*P, R*) is the unique equilibrium, meaning Firm 1 chooses the *P* strategy while Firm 2 opts for the R strategy. All else being equal, the strategic choice for each firm hinges on its initial conditions. A firm with lower initial bargaining power and a larger home market is more inclined to choose the *R* strategy. This is because such a firm has the incentive to improve its bargaining weakness and take advantage of its larger market through a relationship-oriented CSR strategy. Conversely, a firm with higher initial bargaining power and a smaller home market is more likely to choose the *P* strategy. This firm seeks to counteract its market disadvantage and leverage its stronger bargaining position by focusing on a product-oriented CSR strategy.

In situations where $\Delta (v-t)\leq min\{\frac{\Delta \alpha \left(v_{h}\;-v_{l}\right)-\frac{\gamma }{n_{X}},\;}{\alpha_{\text{1}}+\Delta \alpha },\frac{\Delta \alpha \left(v_{h}\;-v_{l}\right)-\frac{\gamma }{n_{Z}},\;}{\alpha_{\text{2}}+\Delta \alpha }\}$, both (*P, R*) and (*R, P*) are equilibria. The result illustrates that firms could coordinate on their CSR strategies in this scenario. The presence of multiple equilibria indicates that neither firm has a clear incentive to unilaterally deviate from its chosen strategy, leading to a situation where both strategies (*P*, *R*) or (*R*, *P*) can coexist as stable outcomes. In essence, the firms might recognize the mutual benefits of avoiding direct competition in the same CSR domain and instead, implicitly or explicitly, coordinate their strategic choices to optimize their value capture. This coordination reflects a balance where each firm finds its chosen strategy advantageous given the strategy of its competitor, leading to a cooperative equilibrium that allows both firms to effectively manage their market segments without eroding each other’s competitive advantages.

**Fig 1:**
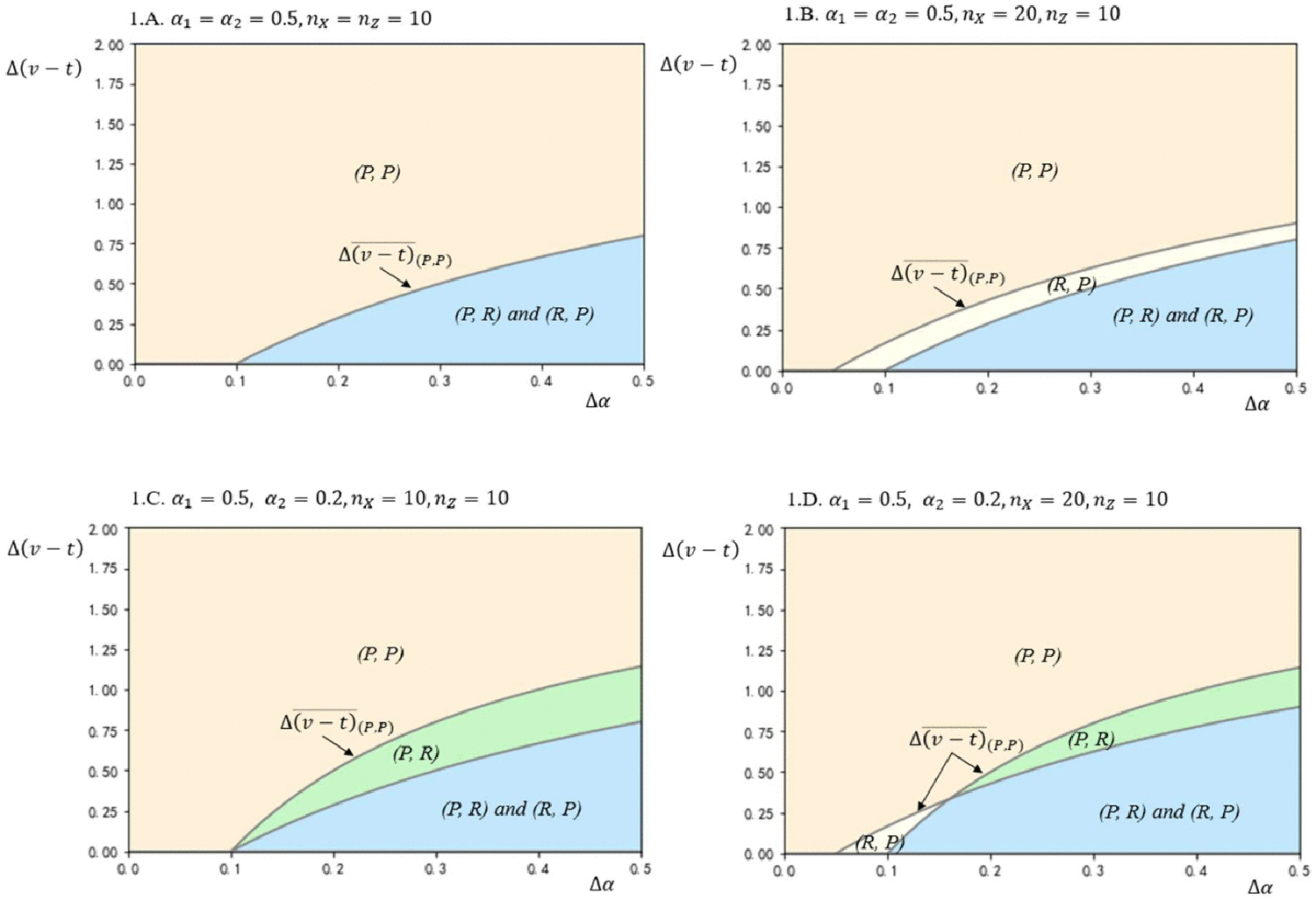
Equilibrium plots in terms of Δ(𝐯−𝐭) and Δα for different values of ${𝐧}_{X},n_{Z},$ $\alpha_{1}\;and\;\alpha_{2}$, based on the following other parameter values: ${𝐧}_{Y}=10,\;v_{h}=4,\;v_{l}=2,\;\gamma =2$.

Fig 1 depicts how firms’ CSR strategy choices vary with the enhancements in bargaining power Δα and the difference between product value increment and transaction costs Δ(v−t). In Panel A, this paper examines a case where the initial conditions for both firms are symmetric, meaning that both firms have equal bargaining power ($\alpha_{\text{1}}=\alpha_{\text{2}}=\text{0}.\text{5}$) and equal home market segment sizes ($n_{X}=n_{Z}=\text{1}\text{0}$). The equilibrium is determined by the values of $\frac{\Delta \alpha \left(v_{h}\;-v_{l}\right)-\frac{\gamma }{n_{X}},\;}{\alpha_{\text{1}}+\Delta \alpha }\text{ and }\frac{\Delta \alpha \left(v_{h}\;-v_{l}\right)-\frac{\gamma }{n_{Z}},\;}{\alpha_{\text{2}}+\Delta \alpha }$, as derived from Proposition 1. Panel A shows that when Δ(v−tis sufficiently high, the symmetric equilibrium (*P, P*) emerges. This implies that both firms find it beneficial to pursue product-oriented CSR strategies under symmetric initial conditions, leading to a situation where product characteristics become the focal point of competition. On the other hand, when Δα is relatively high and Δ(v−t) is relatively low, both asymmetric equilibria (*P*, *R*) and (*R*, *P*) are possible. This indicates that in scenarios where neither the product-oriented nor the relationship-oriented CSR strategy provides a clear competitive advantage, firms may opt for different strategies, resulting in a heterogeneous equilibrium that can generate higher profits for each firm in the industrial setting. The results in Fig 1, Panel A highlight that under symmetric initial conditions, the choice of CSR strategy is heavily influenced by the relative effectiveness of increasing product value versus reducing transaction costs. When both strategies have their respective strengths, firms may strategically diversify their approaches to maximize overall industry profits without eroding each other’s competitive advantages.

Result (ii) of Proposition 1 suggests that a unique equilibrium of strategic heterogeneity is possible under asymmetric initial conditions. In scenarios where firms adopt heterogeneous CSR strategies, one firm focuses on enhancing its value creation through product-oriented CSR characteristics, while the other firm increases its value by strengthening its relationship with buyers. Panel B in Fig 1 illustrates this case in the yellow area, reflecting asymmetric conditions where all parameters are the same as in Panel A, except for the key difference that the initial home market sizes vary across firms ($n_{X}=\text{2}\text{0},\;n_{Z}=\text{1}\text{0}$). With this asymmetry, a unique equilibrium of strategic heterogeneity emerges, where the firm with the larger home market adopts the relationship-oriented CSR strategy, while its rival pursues the product-oriented CSR strategy. This outcome can be understood by examining the marginal returns from increasing levels of product-oriented CSR and relationship-oriented CSR. In a biform game model, value creation and value capture are complementary, contributing to each firm’s returns. If the value created from the *P* strategy is not sufficiently high, the firm with the larger market size benefits more from increasing its relationship with buyers, as this strategy leads to both reduced transaction costs and increased bargaining power.

Panel C in Fig 1 highlights the impact of firms’ asymmetric initial bargaining power by setting the same parameters as in Panel A, with the key difference being that the initial bargaining power varies across firms ($\alpha_{\text{1}}=\text{0}.\text{5},\;\alpha_{\text{2}}=\text{0}.\text{2}$). Under these asymmetric conditions, the model also reveals the possibility of a unique equilibrium of strategic heterogeneity. In this scenario, a green area emerges where the firm with the higher initial bargaining power opts for the product-oriented CSR strategy, while its rival adopts the relationship-oriented CSR strategy. This outcome suggests that the higher a firm’s initial bargaining power, the more it stands to gain from enhancing its product’s CSR characteristics. The results indicate a complementarity between bargaining power and the product-oriented CSR strategy, where stronger bargaining power amplifies the benefits of focusing on product-related CSR efforts. When combining the insights from Panels B and C, this study observes a broader complementarity between value creation and value capture, demonstrating how firms strategically align their CSR strategies to maximize their competitive advantage based on their initial conditions.

In Panel D of Fig 1, this study explores another case of asymmetric conditions, where the parameters from Panel A are adjusted to reflect a larger home market segment size and greater bargaining power for Firm 1 ($n_{X}=\text{2}\text{0},\;n_{Z}=\text{1}\text{0}$, $\alpha_{\text{1}}=\text{0}.\text{5},\;\alpha_{\text{2}}=\text{0}.\text{2}$). This scenario illustrates how the unique equilibrium of CSR strategy choices shifts under these new conditions, especially when there is a high Δα and medium Δ(v−t). Conversely, in the yellow area, if the effectiveness of Firm 2’s *R* strategy is not significant, Firm 1 may also choose the *R* strategy as the value enhancement from the *P* strategy becomes less impactful. In this case, Firm 1’s motivation to choose the *R* strategy stems from its desire to capitalize on its larger home market advantage rather than its bargaining power. The firm aims to strengthen its existing market dominance by improving buyer relationships, rather than focusing on product enhancements that may not provide as much additional value.

Overall, Panel D demonstrates that the choice between product-oriented and relationship-oriented CSR strategies is influenced by the interplay between a firm’s home market size, initial bargaining power, and the effectiveness of each strategy. Firms strategically select their CSR approaches based on their unique market positions and competitive dynamics, with weaker firms seeking to offset their disadvantages and stronger firms looking to leverage their strengths.

**Fig 2:**
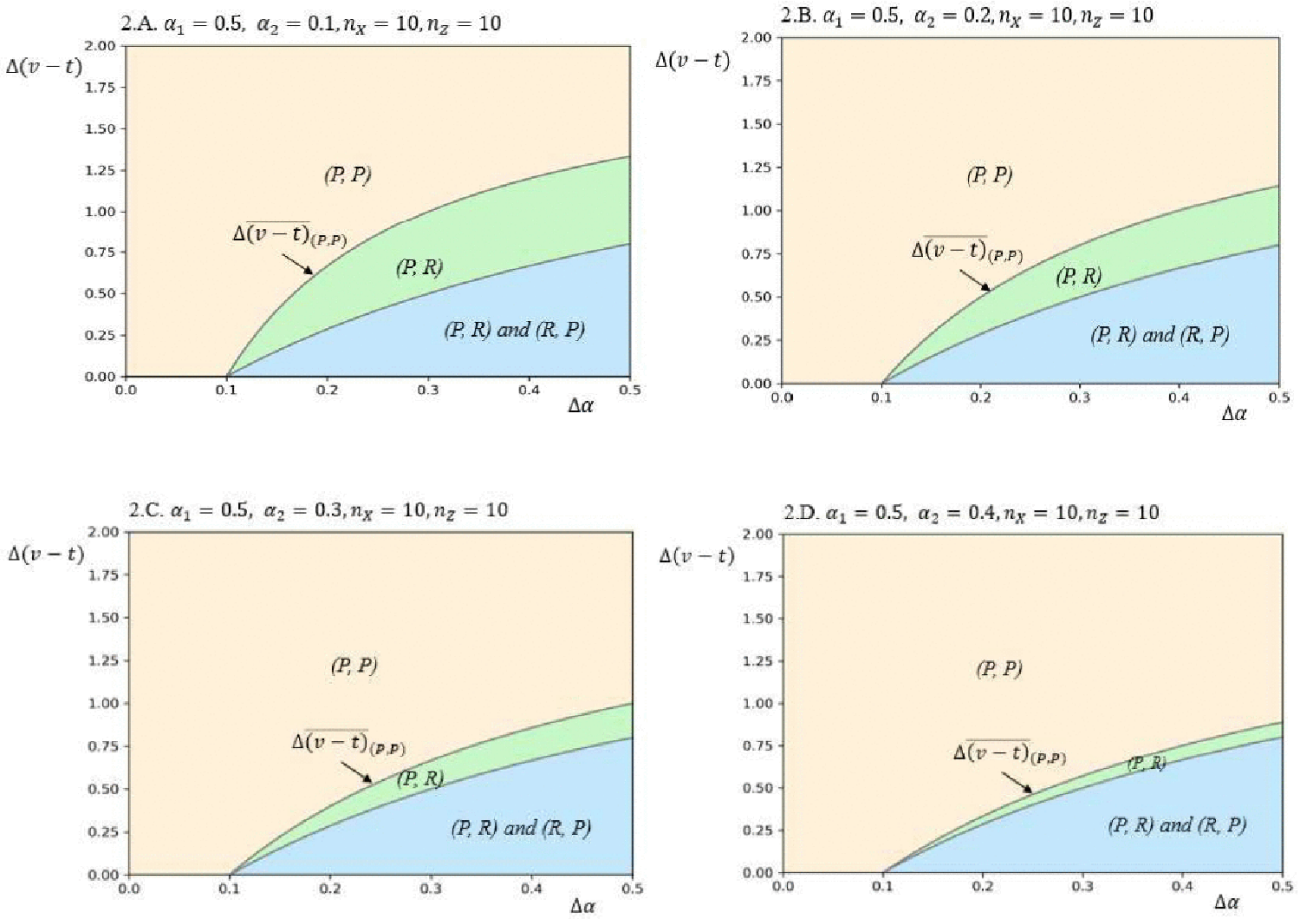
Equilibrium plots in terms of Δ(𝐯−𝐭) and Δα for different values of $\alpha_{\text{2}}$, based on the following other parameter values: ${𝐧}_{X}=10,\;n_{Y}=10,\;n_{Z}=10,\;\alpha_{1}=0.5,\;v_{h}=4,\;v_{l}=2,\;\gamma =2$.

**Fig 3:**
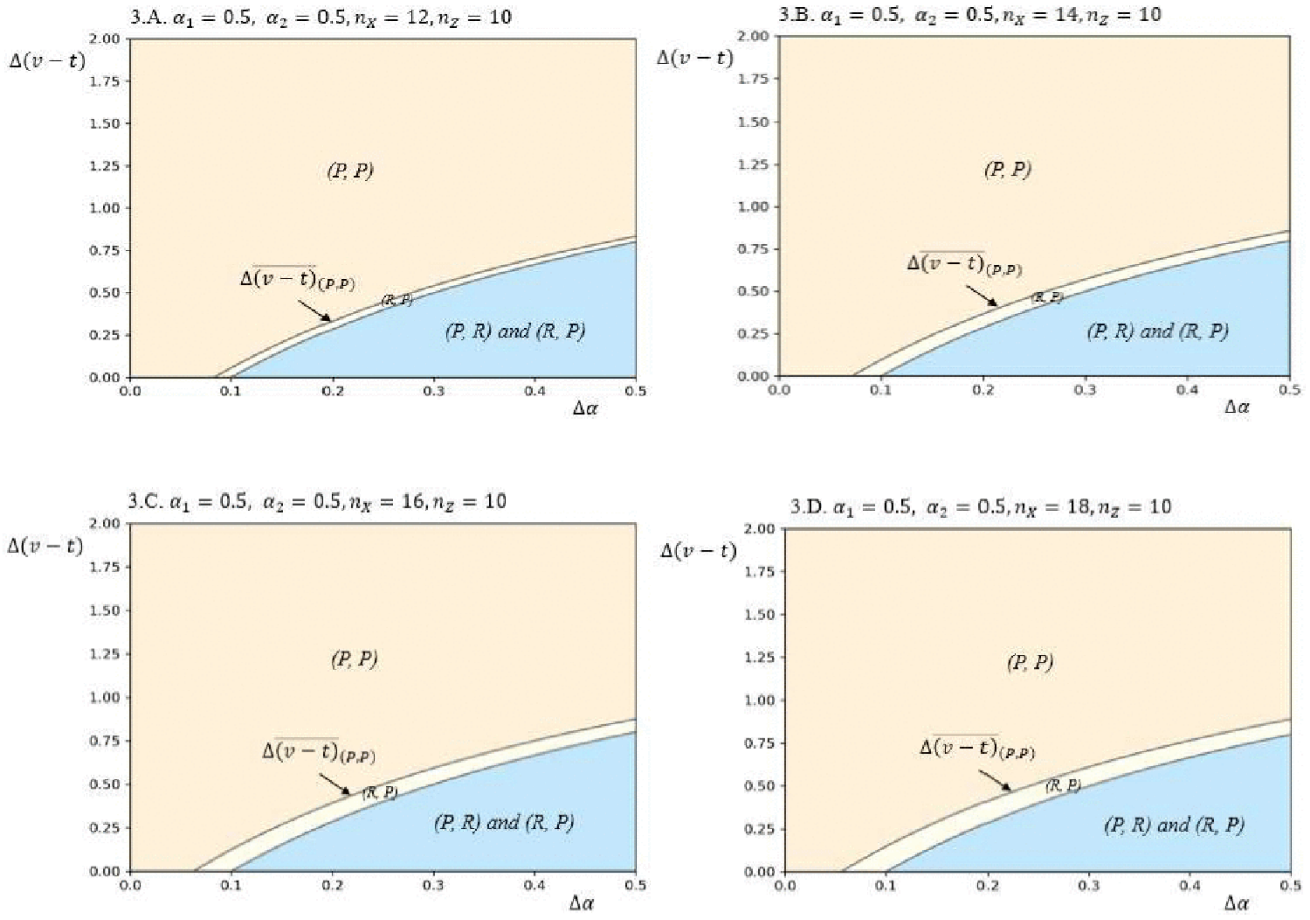
Equilibrium plots in terms of Δ(𝐯−𝐭) and Δα for different values of ${𝐧}_{X}$, based on the following other parameter values: ${𝐧}_{Y}=10,\;n_{Z}=10,\;\alpha_{1}=0.5,\;\alpha_{2}=0.5,\;v_{h}=4,\;v_{l}=2,\;\gamma =2$.

To investigate how variations in key parameters affect model outcomes, this study conducted sensitivity analyses to evaluate the influence of market scale and bargaining power on equilibrium under low information asymmetry, as illustrated in Figs 2 and 3. Fig 2 demonstrates that increasing the asymmetry in bargaining power expands the lower-right section of the yellow area, transitioning from a (*P*, *P*) configuration to (*P*, *R*). This shift indicates that the firm with lower bargaining power adopts the *R* strategy, motivated by the advantages of increased bargaining power in its domestic market, which overcomes its bargaining power weakness. Similarly, Fig 3 shows that a rise in market scale asymmetry increases the lower-right section of the yellow area, transitioning from (*P*, *P*) to (*R*, *P*). In this case, the firm with a larger market scale adopts the *R* strategy, driven by the ability to better align its increased bargaining power with its strengths in the domestic market.

### High information asymmetry industrial context

Proposition 1 identifies three potential configurations of CSR strategies in a low information asymmetry context. The first scenario involves CSR strategy homogeneity, where firms converge on a (*P*, *P*) equilibrium within a specific parameter space. The second scenario reveals a unique strategic heterogeneity, where either a (*P*, *R*) or (*R*, *P*) equilibrium emerges exclusively. The third scenario highlights a parameter space with strategic heterogeneity that allows for multiple equilibria, meaning that both (*P*, *R*) and (*R*, *P*) outcomes are possible. This study now turns to the equilibrium configurations in a high information asymmetry context. In such markets, product-oriented CSR characteristics are less likely to be easily perceived and validated by buyers, while relationship-oriented CSR activities prove more effective in building trust and reducing transaction costs. In this high information asymmetry setting, where Δ(t−v)>0, the model’s point estimates are presented in Table 3.

**Table 3:** The payoff matrix of the game between firms in a high information asymmetry context.

Firm 2 Firm 1	*P*	*R*
*P*	${n_{X}\alpha }_{\text{1}}(v_{h}\;-v_{l})$, ${n_{Z}\alpha }_{\text{2}}(v_{h}\;-v_{l})$	${n_{X}\alpha }_{\text{1}}\left(v_{h}\;-v_{l}+\Delta v-\Delta t\right)$, $\begin{array}{c} {n_{Z}(\alpha }_{\text{2}}+\Delta \alpha )\left(v_{h}\;-v_{l}-\Delta v+\Delta t\right)+n_{Y}{(\alpha }_{\text{2}}+\Delta \alpha )(\Delta t-\Delta v)-\gamma \end{array}$
*R*	$\begin{array}{c} {n_{X}(\alpha }_{\text{1}}+\Delta \alpha )\left(v_{h}\;-v_{l}-\Delta v+\Delta t\right)+n_{Y}{(\alpha }_{\text{1}}+\Delta \alpha )(\Delta t-\Delta v)-\gamma \end{array}$, ${n_{Z}\alpha }_{\text{2}}\left(v_{h}\;-v_{l}+\Delta v-\Delta t\right)$	${n_{X}\alpha }_{\text{1}}\left(v_{h}\;-v_{l}\right)-\gamma$, ${n_{Z}\alpha }_{\text{2}}(v_{h}\;-v_{l})-\gamma$

Based on the payoff matrix, the firms choose their CSR strategies, and the equilibrium is summarized in Proposition 2.

Proposition 2. *Depending on parameters, in a high information asymmetry context, there is either an equilibrium where both firms pursue the same CSR strategy or an equilibrium where one firm pursues a product-oriented CSR strategy, and the other firm pursues a relationship-oriented CSR strategy. Formally*:

(i) *There exists a threshold* $\Delta {\overset{―}{\left(t-v\right)}}_{\left(P,P\right)}=min\{\frac{\gamma -n_{X}\Delta \alpha (v_{h}\;-v_{l}}{\left(n_{X}+n_{Y})(\alpha_{\text{1}}+\Delta \alpha \right)},\frac{\gamma -n_{Z}\Delta \alpha (v_{h}\;-v_{l})\;}{\left(n_{Z}+n_{Y})(\alpha_{\text{2}}+\Delta \alpha \right)}\}\geq \text{0}$ *such that (P, P) is an equilibrium if and only if* $\Delta (t-v)\leq \Delta ({\overset{―}{t-v})}_{\left(P,P\right)}$;
(ii) *There exists another threshold* $\Delta {(\overset{―}{t-v)}}_{\left(R,R\right)}=max\{\frac{\gamma \;}{n_{X}\alpha_{\text{1}}},\frac{\gamma \;}{n_{Z}\alpha_{\text{2}}}\}$ *≥ 0 such that (R, R) is an equilibrium if and only if* $\Delta (t-v)\geq \Delta ({\overset{―}{t-v})}_{\left(R,R\right)}$*.*
(iii) *Either (P, R) or (R, P) is the unique equilibrium if*

$max\{\frac{\gamma -n_{X}\Delta \alpha (v_{h}\;-v_{l}}{\left(n_{X}+n_{Y})(\alpha_{\text{1}}+\Delta \alpha \right)},\frac{\gamma -n_{Z}\Delta \alpha (v_{h}\;-v_{l})\;}{\left(n_{Z}+n_{Y})(\alpha_{\text{2}}+\Delta \alpha \right)}\}\geq \Delta (t-v)\geq min\{\frac{\gamma -n_{X}\Delta \alpha (v_{h}\;-v_{l})\;}{\left(n_{X}+n_{Y})(\alpha_{\text{1}}+\Delta \alpha \right)},\frac{\gamma -n_{Z}\Delta \alpha (v_{h}\;-v_{l})\;}{\left(n_{Z}+n_{Y})(\alpha_{\text{2}}+\Delta \alpha \right)}\}$ or $max\{\frac{\gamma \;}{n_{X}\alpha_{\text{1}}},\frac{\gamma \;}{n_{Z}\alpha_{\text{2}}}\}$. $\geq \Delta (t-v)\geq min\{\frac{\gamma \;}{n_{X}\alpha_{\text{1}}},\frac{\gamma \;}{n_{Z}\alpha_{\text{2}}}\}$.

(iv) *Both (P, R) and (R, P) are a possible equilibria if and only if* $max\{\frac{\gamma -n_{X}\Delta \alpha (v_{h}\;-v_{l}}{\left(n_{X}+n_{Y})(\alpha_{\text{1}}+\Delta \alpha \right)},\frac{\gamma -n_{Z}\Delta \alpha (v_{h}\;-v_{l})\;}{\left(n_{Z}+n_{Y})(\alpha_{\text{2}}+\Delta \alpha \right)}\}\leq \Delta (t-v)\leq min\{\frac{\gamma \;}{n_{X}\alpha_{\text{1}}},\frac{\gamma \;}{n_{Z}\alpha_{\text{2}}}\}$.

Results (i) and (ii) establish the conditions under which firms in the model opt for homogeneous strategies in a high information asymmetry context. The findings indicate that both firms will choose a product-oriented CSR strategy when the relative returns from the relationship-oriented strategy, represented by Δ(t−vand Δα, are sufficiently low. Conversely, when these relative returns are high, both firms are inclined to adopt a relationship-oriented CSR strategy. In this scenario, neither firm is motivated to switch from an *R* strategy to a *P* strategy, as doing so would result in a loss of value.

In some instances where $max\{\frac{\gamma -n_{X}\Delta \alpha (v_{h}\;-v_{l}}{\left(n_{X}+n_{Y})(\alpha_{\text{1}}+\Delta \alpha \right)},\frac{\gamma -n_{Z}\Delta \alpha (v_{h}\;-v_{l})\;}{\left(n_{Z}+n_{Y})(\alpha_{\text{2}}+\Delta \alpha \right)}\}\geq \Delta (t-v)\geq min\{\frac{\gamma -n_{X}\Delta \alpha (v_{h}\;-v_{l})\;}{\left(n_{X}+n_{Y})(\alpha_{\text{1}}+\Delta \alpha \right)},\frac{\gamma -n_{Z}\Delta \alpha (v_{h}\;-v_{l})\;}{\left(n_{Z}+n_{Y})(\alpha_{\text{2}}+\Delta \alpha \right)}\}$ or $max\{\frac{\gamma \;}{n_{X}\alpha_{\text{1}}},\frac{\gamma \;}{n_{Z}\alpha_{\text{2}}}\}$. $\geq \Delta (t-v)\geq min\{\frac{\gamma \;}{n_{X}\alpha_{\text{1}}},\frac{\gamma \;}{n_{Z}\alpha_{\text{2}}}\}$, this study finds a unique asymmetric equilibrium where firms choose either (*P*, *R*) or (*R*, *P*). The outcome of this strategic asymmetry primarily depends on the size of the firms’ home markets and their initial bargaining power. In situations where $max\{\frac{\gamma -n_{X}\Delta \alpha (v_{h}\;-v_{l}}{\left(n_{X}+n_{Y})(\alpha_{\text{1}}+\Delta \alpha \right)},\frac{\gamma -n_{Z}\Delta \alpha (v_{h}\;-v_{l})\;}{\left(n_{Z}+n_{Y})(\alpha_{\text{2}}+\Delta \alpha \right)}\}\leq \Delta (t-v)\leq min\{\frac{\gamma \;}{n_{X}\alpha_{\text{1}}},\frac{\gamma \;}{n_{Z}\alpha_{\text{2}}}\}$, there are possibilities for two asymmetric equilibria, allowing firms to coordinate their strategy choices on either (*P*, *R*) or (*R*, *P*), with both being valid equilibria.

**Fig 4:**
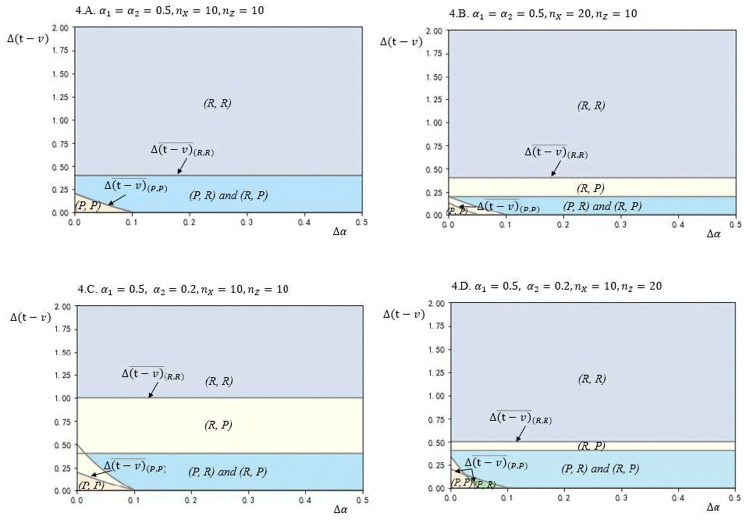
Equilibrium plots in terms of Δ(𝐭−𝐯) and Δα for different values of ${𝐧}_{X,}\;n_{Z,}$ $\alpha_{1}\;and\;\alpha_{2}$, based on the following other parameter values: ${𝐧}_{Y}=10,\;v_{h}=\text{4},\;v_{l}=2,\;\gamma =2$.

Fig 4 illustrates how firms’ CSR strategy choices vary with the parameter of bargaining power enhancements Δα and the difference between the transaction cost reduction and the product value increase Δ(t−v). Panel A presents a case where the firms’ initial conditions are symmetric, both in terms of their bargaining power ($\alpha_{\text{1}}=\alpha_{\text{2}}=\text{0}.\text{5}$) and home market segment sizes ($n_{X}=n_{Z}=\text{1}\text{0}$). The equilibrium is determined by the value of $\frac{\gamma -n_{X}\Delta \alpha (v_{h}\;-v_{l}}{\left(n_{X}+n_{Y})(\alpha_{\text{1}}+\Delta \alpha \right)}$, $\frac{\gamma -n_{Z}\Delta \alpha (v_{h}\;-v_{l}}{\left(n_{Z}+n_{Y})(\alpha_{\text{2}}+\Delta \alpha \right)}$, $\frac{\gamma \;}{n_{X}\alpha_{\text{1}}}$, and $\frac{\gamma \;}{n_{Z}\alpha_{\text{2}}}$ as outlined in Proposition 2. Panel A indicates that, under symmetric initial conditions, both asymmetric equilibria (*P*, *R*) and (*R*, *P*) can occur when Δα is relatively high and Δ(t−v) is relatively low.

Result (iii) of Proposition 2 suggests that a unique equilibrium of strategic heterogeneity can emerge under asymmetric initial conditions. Panels B and C in Fig 4 highlight the heterogeneous CSR strategy configurations within the yellow areas. In Panel B, firms differ in their initial home market sizes ($n_{X}=\text{2}\text{0},\;n_{Z}=\text{1}\text{0}$), while all other parameters remain consistent with Panel A. In Panel C, firms vary in their initial bargaining power ($\alpha_{\text{1}}=\text{0}.\text{5},\;\alpha_{\text{2}}=\text{0}.\text{2}$), again with all other parameters the same as in Panel A. This asymmetry allows for the realization of a unique equilibrium in CSR strategy heterogeneity. In both Panels B and C, the firm with either the larger home market or greater bargaining power opts for the *R* strategy, while its rival adopts the *P* strategy in the yellow regions. In Panel B, the complementarity between market size and bargaining power is evident: the larger a firm’s market size, the greater the returns from strengthening its relationship with buyers. In Panel C, the firm with higher initial bargaining power also chooses the *R* strategy, leveraging its position to enhance trust with buyers. By combining the insights from Panels B and C, this study observes a clear incentive for firms to capitalize on their respective strengths in determining their CSR strategies.

The paper illustrates a scenario where Firm 2 has a larger home market segment but lower bargaining power ($n_{X}=\text{1}\text{0},\;n_{Z}=\text{2}\text{0}$, $\alpha_{\text{1}}=\text{0}.\text{5},\;\alpha_{\text{2}}=\text{0}.\text{2}$). In Panel D, the green region indicates that when both Δ(t−v) and Δα are low, the firm with the larger home market and lower initial bargaining power (Firm 2) may opt for the *R* strategy. This choice is driven by the motivation to compensate for its weaker bargaining position. The results in Fig 4 highlight the paradox firms face in their CSR strategy decisions: balancing the need to leverage their strengths while simultaneously addressing their weaknesses.

**Fig 5:**
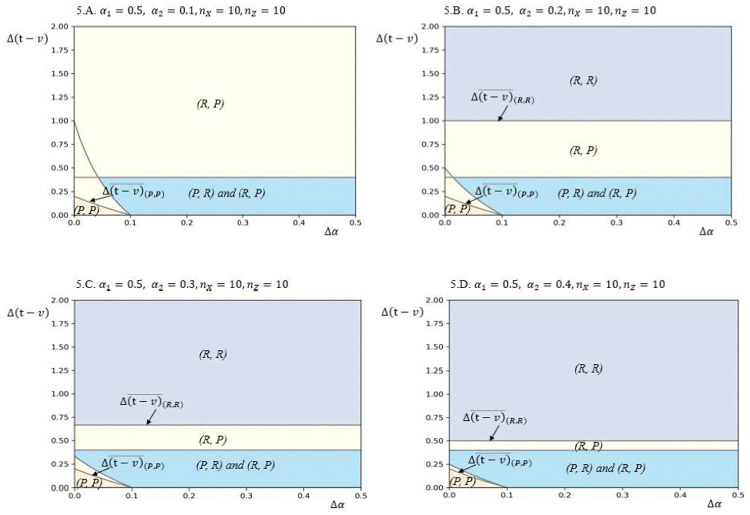
Equilibrium plots in terms of Δ(𝐭−𝐯) and Δα for different values of $\alpha_{\text{2}}$, based on the following other parameter values: ${𝐧}_{X}=10,\;n_{Y}=10,\;n_{Z}=10,\;\alpha_{1}=0.5,\;v_{h}=4,\;v_{l}=2,\;\gamma =2$.

**Fig 6:**
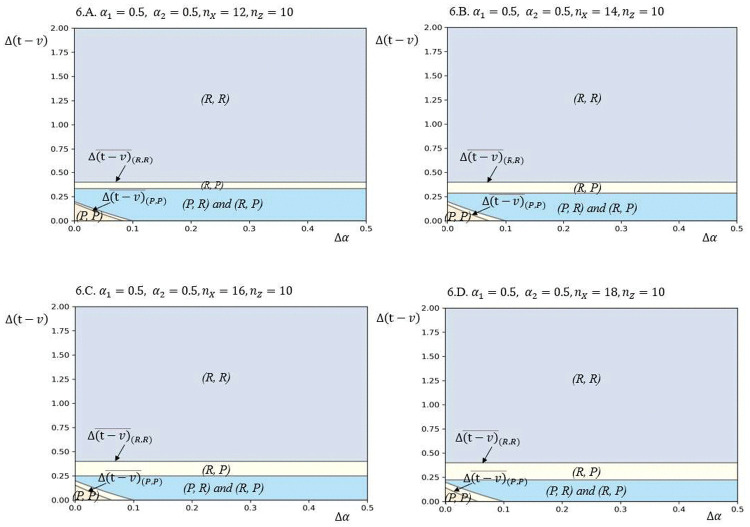
Equilibrium plots in terms of Δ(𝐭−𝐯) and Δα for different values of ${𝐧}_{X}$, based on the following other parameter values: ${𝐧}_{Y}=10,\;n_{Z}=10,\;\alpha_{1}=0.5,\;\alpha_{2}=0.5,v_{h}=4,\;v_{l}=2,\;\gamma =2$.

To examine the impact of key parameter variations on model outcomes, this study conducted sensitivity analyses, focusing on the effects of market scale and bargaining power on equilibrium under high information asymmetry, as shown in Figs 5 and 6. Fig 5 illustrates that increasing asymmetry in bargaining power reduces the region of (*R*, *R*) configuration while expanding the (*R*, *P*) configuration, suggesting that firms with lesser bargaining power are more likely to adopt a *P* strategy. This shift is driven by a reduction in the fixed costs associated with the *R* strategy when firms have limited bargaining power. Similarly, Fig 6 shows that greater asymmetry in market scale enlarges the (*R*, *P*) configuration, with firms possessing larger market scales favoring the *R* strategy. This shift reflects the advantage of leveraging the *R* strategy, which better aligns with the strengths conferred by their market scale.

In summary, this section analyzes equilibria of CSR strategies under rivalry. It finds that strategic equilibria in CSR strategies are influenced by key factors including information asymmetry, product value, market segment size, transaction costs, and bargaining power, with firms tending to adopt product- or relationship-oriented approaches when the derived value from each is substantially high. Firms operating in larger market sizes consistently lean toward product-oriented CSR strategies to optimize their competitive positioning. In contexts of low information asymmetry, firms possessing lower bargaining power are more likely to select relationship-oriented strategies, while under high information asymmetry, these same firms prefer product-oriented strategies.

## Value capture and industry value creation

In this section, this paper analyzes the impact of firms’ CSR strategy choices on overall industry value creation and the distribution of that value among firms and buyers. First, it examines value distribution when firms coordinate their CSR strategies, either focusing on product-oriented or relationship-oriented activities. It then analyzes how different parameters affect value capture by firms and buyers, considering not only the effects within a given equilibrium configuration but also the potential transitions between different equilibria. A key focus is on the significant influence of these parameters on CSR strategy choices and their resulting outcomes.

Considering different levels of information asymmetry, this study evaluates the value capture by firms, buyers, and all players. When firms can coordinate on either product-oriented or relationship-oriented CSR strategies, the difference between $\Pi_{\text{1}}+\Pi_{\text{2}}|(P,R)$ and ${\mathrm{\backslash itPi}}_{\text{1}}+\Pi_{\text{2}}|(R,P)$ is $\Delta \left({\mathrm{\backslash itPi}}_{\text{1}}+{\mathrm{\backslash itPi}}_{\text{2}}\right)=\left({n_{X}\alpha }_{\text{1}}-{n_{Z}\alpha }_{\text{2}}\right)(\Delta v-\Delta t)+\Delta \alpha \left(n_{Z}-n_{X}\mathrm{\backslash rightleft}(v_{h}\;-v_{l}-\Delta v+\Delta t\right)+{n_{Y}(\alpha }_{\text{1}}-\alpha_{\text{2}})(\Delta v-\Delta t)$. The difference of U|(P,R) and U|(P,R) is $\Delta U=\text{2}n_{X}(\Delta v-\Delta t)+\text{2}n_{Z}(\Delta t-\Delta v)-\Delta \left({\mathrm{\backslash itPi}}_{\text{1}}+{\mathrm{\backslash itPi}}_{\text{2}}\right)$. Therefore, this study has the following:

Proposition 3. *When (P, R) and (R, P) are both equilibrium strategies*:

(i) ***Firms’ Total Value Capture*** *: Given a specific set of parameters, the total value captured by firms can be higher if they coordinate their strategic choices by alternating between (P, R) and (R, P). The optimal combination of these strategies is influenced by factors such as the size of segmented markets and the firms’ initial bargaining power.*
(ii) ***Buyers’ Value Capture*** *: Given a set of parameters, expected buyers’ value capture could be lower when firms coordinate on (P, R) and (R, P) strategies.*

Result (i) suggests that firms’ total value capture is more likely to be higher when the firm with higher initial bargaining power chooses the product-oriented CSR strategy while its rival adopts the relationship-oriented strategy, particularly when the three market segments are of equal size in a low information asymmetry context. Conversely, in a high information asymmetry context, firms’ total value capture is more likely to be higher when the firm with higher initial bargaining power chooses the relationship-oriented strategy and its rival adopts the product-oriented strategy. Result (ii) indicates that buyers’ total value capture tends to be lower when firms can coordinate on the (*P*, *R*) and (*R*, *P*) strategies. Given a fixed level of total industry value creation, the increase in firms’ value capture corresponds to a decrease in the value captured by buyers.

**Fig 7:**
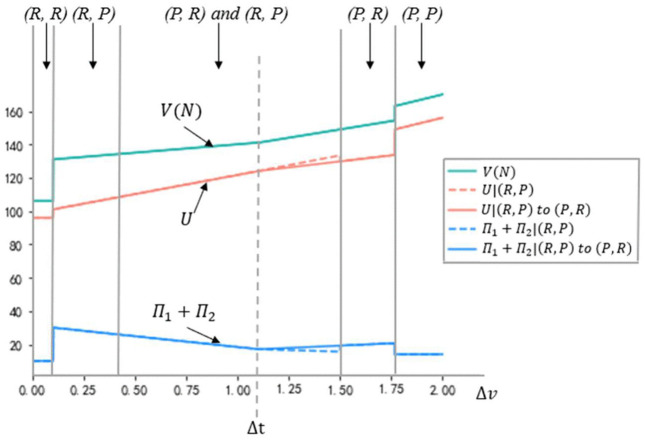
Firms’ value capture ${𝛱}_{1}+{𝛱}_{2}$ and buyers’ value capture U with shifts in Δ𝐯, based on the following other parameter values: ${𝐯}_{h}=4,\;v_{m}=3,\;v_{l}=2,\;n_{X}=10,\;n_{Y}=10,\;n_{Z}=10,\;\alpha_{1}=0.5,\;\alpha_{2}=0.2,\;\gamma =2,\;\Delta \alpha =0.25,\;\Delta t=1.1$.

To illustrate the impact of changes in the value creation enhancement Δv on the expected total value capture of the industry, firms, and buyers, Fig 7 presents the results of Proposition 3. The Fig highlights a region where both (*P*, *R*) and (*R*, *P*) are equilibrium strategies. In that region, expected firm value capture is higher when the strategic combination is (*R*, *P*) if Δv<Δt and (*P, R*) if Δv≥Δt, as shown by the solid blue line. Conversely, buyers’ total value capture is lower in this scenario, as indicated by the solid red line.

Fig 7 also highlights the importance of considering both local and transition effects of parameter shifts. Within regions of homogeneous strategic equilibrium, such as (*P*, *P*) or (*R*, *R*), increases in Δv do not impact firm value capture. However, within regions of heterogeneous strategic equilibrium, such as (*R*, *P*) or (*P*, *R*), increases in Δv may either raise or reduce firm value capture. Overall, an increase in Δv enhances buyer value capture and industry value creation. Additionally, an increase in Δv that triggers a transition from a homogeneous strategy equilibrium to a heterogeneous one, or vice versa (e.g., from (*R*, *R*) to (*R*, *P*) or from (*P*, *R*) to (*P*, *P*)), results in a discrete change—either a fall or an increase—in firm value capture, buyer value capture, and industry value creation.

This paper now characterizes how value capture by firms and buyers, as well as overall industry value creation, vary with the parameters in the model. It focuses on the parameters that determine the effectiveness of the two CSR strategies: their potential enhancements in value and bargaining power (Δv, Δα, and Δt). Additionally, this study considers the effect of the initial level of home-segment value creation ($v_{h}$, $v_{m}$, and $v_{l}$). Then, it presents the following proposition:

Proposition 4:

(i) *Firms’ value capture* $\Pi_{\text{1}}+\Pi_{\text{2}}|(s_{i}^{*},s_{-i}^{*})$ *increases with* Δα *and is nonmonotonic with respect to* Δv *and* Δt*. Buyers’ value capture* $U|(s_{i}^{*},s_{-i}^{*})$ *decreases with* Δα *and is also nonmonotonic with respect to* Δv *and* Δt*. Overall industry value creation increases with both* Δv *and* Δt*.*
(ii) *Firms’ value capture increases with* $v_{h}-v_{l}$*. Buyers’ value capture* $U|(s_{i}^{*},s_{-i}^{*})$ *increases with* $v_{m}$ *but decreases with* $v_{h}-v_{l}$*. Industry value creation V(N)* $\left|(s_{i}^{*},s_{-i}^{*}\right)$ *increases with* $v_{h}$*,* $v_{m}$*, and* $v_{l}$*.*

In Proposition 4 (i), this study asserts that the parameters determining the effectiveness of the *P* and *R* strategies—Δv,  Δα, and Δt —have significant impacts on firm value capture. Specifically, within heterogeneous strategy equilibria, an increase in the effectiveness of a firm’s chosen strategy (Δα and Δt for *R* strategy, Δv for *P* strategy) benefits the firm. Conversely, an increase in the effectiveness of the competing strategy (Δv for *R* strategy or Δt for *P* strategy) reduces the attractiveness of the firm’s current strategy. Additionally, increases in the effectiveness of either the *P* or *R* strategy may enhance a firm’s expected value capture through transition effects. These occur by making another strategic equilibrium relatively more attractive, prompting either the rival firm or both firms to shift their CSR strategies.

**Fig 8:**
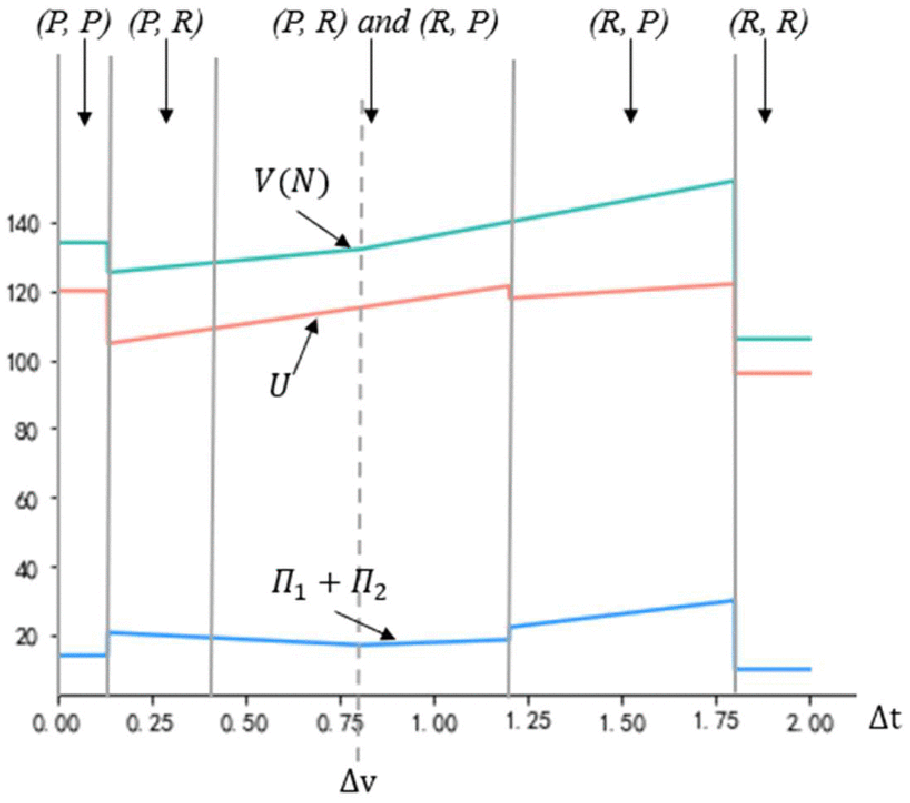
Industry value creation *V(N)*, firms’ value capture ${𝛱}_{1}+{𝛱}_{2},$ and buyers’ value capture U with shifts in Δ𝐭, based on the following other parameter values: ${𝐯}_{h}=4,\;v_{m}=3,\;v_{l}=2,\;n_{X}=10,\;n_{Y}=10,\;n_{Z}=10,\;\alpha_{1}=0.5,\;\alpha_{2}=0.2,\;\gamma =2,\;\Delta \alpha =0.25,\;\Delta v=0.8$.

Fig 8 illustrates the impact of Δt on the firms’ value capture, buyers’ value capture, and industry value creation. The effects of Δt on value creation and capture are qualitatively similar to those of Δv shown in Fig 8, including the various transition effects. However, a transition to the (*R*, *R*) equilibrium leads to a decline in firms’ value capture, buyers’ value capture, and industry value creation. This decline occurs because, when both firms adopt the *R* strategy, they fail to establish a differential relationship pattern, leading to no reduction in transaction costs and no increase in bargaining power.

Changes in the effectiveness of CSR strategies also impact buyers’ value capture. As the effectiveness of the *R* strategy increases, the expected share of added value that firms capture through this strategy rises, while the likelihood of firms choosing the *P* strategy decreases. This shift negatively affects expected buyers’ value capture. However, increases in the effectiveness of the *R* strategy positively impact total industry value creation until both firms opt for the *R* strategy. Conversely, increases in the effectiveness of the *P* strategy have a nonmonotonic effect on firm value capture, leading to a nonmonotonic impact on expected buyers’ value capture, although the overall impact on total industry value creation remains positive.

Result (ii) of Proposition 4 addresses the impact of the initial home-segment competitive advantage $v_{h}-v_{l}$. This study finds that $v_{h}-v_{l}$ positively influences expected firm value capture by contributing to the increase in firms’ added value to their coalitions. In contrast, buyers’ value capture decreases as $v_{h}-v_{l}$ increases but rises with $v_{m}$. Finally, this paper observes that increases in $v_{h}-v_{l}$ have a non-monotonic effect on industry value creation, while industry value creation consistently increases with higher $v_{h}$, $v_{m}$, and $v_{l}$.

## Extension: Fitness of CSR and business strategies

This study now extends the baseline model by incorporating firms’ business strategies. Specifically, instead of assuming firms have no differences in their business strategies, the study considers firms’ business strategies and positioning within segmented markets. This extension contributes to the existing literature on strategy by exploring the connection between CSR strategy and business strategy [50,51]. The findings demonstrate that firms seek to shape their CSR strategies to align with their business strategies while operating within the constraints of the industrial structure [52].

### Model specification

In the extended model, Firm 1 employs a differentiation strategy targeting a niche market, while Firm 2 adopts a cost leadership strategy aimed at capturing a larger mass market. Let *N* be the set of all players—specifically, the two firms and the *n* buyers. Buyers belong to one of three market segments, indexed by *m* = *X*, *Y, Z*. Segment *X* represents a small niche market that prefers differentiated products, while Segment *Z* corresponds to the mass market, which is inclined toward low-priced products. Segment *Y* is a mutable market positioned between the niche and mass markets. In Segment *Y*, a coalition between either firm creates the same value, representing buyers who are easily influenced by the firms’ CSR strategy choices. Buyers are identical within each segment but differ across segments. Let $n_{m}>\text{1}$ denote the number of buyers in segment *m*, such that $n_{X}+n_{Y}+n_{Z}\;=\;n\;$and $n_{X},\;n_{Y}<n_{Z}$. Additionally, let $p\;=\frac{n_{Z}}{n}>\text{0}.\text{5}$ be the fraction of buyers in segment *Z*, where p∈(0.5, 1).

This paper specifically assumes that each firm has a home market segment where it holds an initial competitive advantage (or positive added value). However, neither firm possesses a competitive advantage in the mutable market *Y*:


$$v_{\text{1}X}^{\text{0}}\;=v+v_{h},\;v_{\text{1}Z}^{\text{0}}=\;v-v_{h},\;v_{\text{2}X}^{\text{0}}=v+v_{l},\;v_{\text{2}Z}^{\text{0}}=v-v_{l},v_{\text{1}Y}^{\text{0}}\;=v_{\text{2}Y}^{\text{0}}=v$$ (4)

That is, Firm 1’s home segment is *X*, where its added value for each buyer is ${AV}_{\text{1}X}=v_{h}\;-v_{l}\;>\text{0}$. Similarly, Firm 2’s home segment is *Z*, where its added value for each buyer is ${AV}_{\text{2}Z}=v_{h}\;-v_{l}>\text{0}$. In the mutable market segment *Y*, both Firm 1 and Firm 2 provide the same value, with added values ${AV}_{\text{1}Z}={AV}_{\text{2}Z}=\text{0}$. This indicates that initially, each firm has added value only within its home segment. The value generated by either the *P* strategy or *R* strategy is less than the value difference between a firm’s home market and its rival’s home market:$v_{h}\;-v_{l}>\Delta v,\;\Delta t$. CSR activities could not enable firms to evade into other firms’ home market. However, they could help a firm to take over the mutable market and capture value from the coalition [53].

Furthermore, the effectiveness of the product-oriented CSR strategy varies between the firms. For a firm employing a differentiation strategy, the *P* strategy typically fits well with its business approach. As a result, the value generated by Firm 1 through the *P* strategy is greater than that of Firm 2 by $\Delta v^{\prime }$. Based on the extended model’s specifications, in a low information asymmetry context, this paper derives the payoff matrix shown in Table 4 and presents Proposition 5 as follows.

**Table 4:** The payoff matrix of the game between firms of extended model in a low information asymmetry context.

Firm 2Firm 1	*P*	*R*
*P*	${n_{X}\alpha }_{\text{1}}\left(v_{h}\;-v_{l}+\Delta v^{\prime }\right)+{n_{Y}\alpha }_{\text{1}}\Delta v^{\prime }$, ${n_{Z}\alpha }_{\text{2}}(v_{h}\;-v_{l}-\Delta v^{\prime })$	${n_{X}\alpha }_{\text{1}}\left(v_{h}\;-v_{l}+\Delta v^{\prime }+\Delta v-\Delta t\right)+{n_{Y}\alpha }_{\text{1}}(\Delta v^{\prime }+\Delta v-\Delta t)$, $\begin{array}{c} {n_{Z}(\alpha }_{\text{2}}+\Delta \alpha )\left(v_{h}\;-v_{l}-\Delta v-\Delta v^{\prime }+\Delta t\right)-\gamma \end{array}$
*R*	$\begin{array}{c} {n_{X}(\alpha }_{\text{1}}+\Delta \alpha )\left(v_{h}\;-v_{l}-\Delta v+\Delta t\right)-\gamma \end{array}$, ${n_{Z}\alpha }_{\text{2}}\left(v_{h}\;-v_{l}+\Delta v-\Delta t\right)+{n_{Y}\alpha }_{\text{2}}(\Delta v-\Delta t)$	${n_{X}\alpha }_{\text{1}}\left(v_{h}\;-v_{l}\right)-\gamma$, ${n_{Z}\alpha }_{\text{2}}(v_{h}\;-v_{l})-\gamma$

Proposition 5. *Depending on parameters, in a low information asymmetry context, there is only one symmetric equilibrium where both firms choose the product-oriented CSR strategy, and two asymmetric equilibria where one firm chooses product-oriented CSR strategy, and the other firm chooses relationship-oriented CSR strategy. Formally*:

(i) *There exists a threshold* $\Delta {\overset{―}{\left(v-t\right)}}_{\left(P,P\right)}=max\{\frac{\Delta \alpha \left(v_{h}\;-v_{l}\right)-\frac{\gamma }{n_{X}}-(\text{1}+\frac{n_{Y}}{n_{X}})\alpha_{\text{1}}\Delta v^{\prime }\;}{\alpha_{\text{1}}+\Delta \alpha },\frac{\Delta \alpha \left(v_{h}\;-v_{l}-\Delta v^{\prime }\right)-\frac{\gamma }{n_{Z}},\;}{\alpha_{\text{2}}+\Delta \alpha }\}\geq \text{0}$ *such that (P, P) is an equilibrium if and only if* $\Delta (v-t)\geq \Delta ({\overset{―}{v-t})}_{\left(P,P\right)}$;
(ii) *Either (P, R) or (R, P) is the unique equilibrium if and only if* $min\{\frac{\Delta \alpha \left(v_{h}\;-v_{l}\right)-\frac{\gamma }{n_{X}}-(\text{1}+\frac{n_{Y}}{n_{X}})\alpha_{\text{1}}\Delta v^{\prime }\;}{\alpha_{\text{1}}+\Delta \alpha },\frac{\Delta \alpha \left(v_{h}\;-v_{l}-\Delta v^{\prime }\right)-\frac{\gamma }{n_{Z}},\;}{\alpha_{\text{2}}+\Delta \alpha }\}\leq \Delta (v-t)\leq max\{\frac{\Delta \alpha \left(v_{h}\;-v_{l}\right)-\frac{\gamma }{n_{X}}-(\text{1}+\frac{n_{Y}}{n_{X}})\alpha_{\text{1}}\Delta v^{\prime }\;}{\alpha_{\text{1}}+\Delta \alpha },\frac{\Delta \alpha \left(v_{h}\;-v_{l}-\Delta v^{\prime }\right)-\frac{\gamma }{n_{Z}},\;}{\alpha_{\text{2}}+\Delta \alpha }\}$.
(iii) *Either (P, R) or (R, P) is a possible equilibrium if* $\Delta (v-t)\leq min\{\frac{\Delta \alpha \left(v_{h}\;-v_{l}\right)-\frac{\gamma }{n_{X}}-(\text{1}+\frac{n_{Y}}{n_{X}})\alpha_{\text{1}}\Delta v^{\prime }\;}{\alpha_{\text{1}}+\Delta \alpha },\frac{\Delta \alpha \left(v_{h}\;-v_{l}-\Delta v^{\prime }\right)-\frac{\gamma }{n_{Z}},\;}{\alpha_{\text{2}}+\Delta \alpha }\}$.

Next, this study characterizes the equilibrium configurations under a high information asymmetry setting. The corresponding payoff matrix is presented in Table 5, and Proposition 6 is as follows.

**Table 5:** The payoff matrix of the game between firms of extended model in a high information asymmetry context.

Firm 2Firm 1	*P*	*R*
*P*	${n_{X}\alpha }_{\text{1}}\left(v_{h}\;-v_{l}+\Delta v^{\prime }\right)+{n_{Y}\alpha }_{\text{1}}\Delta v^{\prime }$, ${n_{Z}\alpha }_{\text{2}}(v_{h}\;-v_{l}-\Delta v^{\prime })$	${n_{X}\alpha }_{\text{1}}\left(v_{h}\;-v_{l}+\Delta v+\Delta v^{\prime }-\Delta t\right)$, $\begin{array}{c} {n_{Z}(\alpha }_{\text{2}}+\Delta \alpha )\left(v_{h}\;-v_{l}-\Delta v-\Delta v^{\prime }+\Delta t\right)+n_{Y}{(\alpha }_{\text{2}}+\Delta \alpha )(\Delta t-\Delta v-\Delta v^{\prime })-\gamma \end{array}$
*R*	$\begin{array}{c} {n_{X}(\alpha }_{\text{1}}+\Delta \alpha )\left(v_{h}\;-v_{l}-\Delta v+\Delta t\right)+n_{Y}{(\alpha }_{\text{1}}+\Delta \alpha )(\Delta t-\Delta v)-\gamma \end{array}$, ${n_{Z}\alpha }_{\text{2}}\left(v_{h}\;-v_{l}+\Delta v-\Delta t\right)$	${n_{X}\alpha }_{\text{1}}\left(v_{h}\;-v_{l}\right)-\gamma$, ${n_{Z}\alpha }_{\text{2}}(v_{h}\;-v_{l})-\gamma$

Proposition 6. *Depending on parameters, in a high information asymmetry context, there is either a symmetric equilibrium where both firms choose the same CSR strategy, or an asymmetric equilibrium where one firm chooses a product-oriented CSR strategy and the other firm chooses a relationship-oriented CSR strategy. Formally*:

(i) *There exists a threshold* $\Delta {\overset{―}{\left(t-v\right)}}_{\left(P,P\right)}=min\{\frac{\gamma -n_{X}\Delta \alpha \left(v_{h}\;-v_{l}\right)+\alpha_{\text{1}}\Delta v^{\prime }(n_{X}+n_{Y}}{\left(n_{X}+n_{Y})(\alpha_{\text{1}}+\Delta \alpha \right)},\frac{\gamma -n_{Z}\Delta \alpha \left(v_{h}\;-v_{l}\right)+n_{Z}\Delta \alpha \Delta v^{\prime }+n_{Y}(\alpha_{\text{2}}+\Delta \alpha )\Delta v^{\prime }\;}{\left(n_{Z}+n_{Y})(\alpha_{\text{2}}+\Delta \alpha \right)}\}\geq \text{0}$ *such that  (P, P) is an equilibrium if and only if* $\Delta (t-v)\leq \Delta ({\overset{―}{t-v})}_{\left(P,P\right)}$;
(ii) *There exists another threshold* $\Delta {(\overset{―}{t-v)}}_{\left(R,R\right)}=max\{\frac{\gamma +n_{X}\alpha_{\text{1}}\Delta v^{\prime }\;}{n_{X}\alpha_{\text{1}}},\frac{\gamma \;}{n_{Z}\alpha_{\text{2}}}\}$ *≥ 0 such that (R, R) is an equilibrium if and only if* $\Delta (t-v)\geq \Delta ({\overset{―}{t-v})}_{\left(R,R\right)}$*.*
(iii) *Either (P, R) or (R, P) is the unique equilibrium if*

$\begin{array}{c} max\{\frac{\gamma -n_{X}\Delta \alpha \left(v_{h}\;-v_{l}\right)+\alpha_{\text{1}}\Delta v^{\prime }(n_{X}+n_{Y})\;}{\left(n_{X}+n_{Y})(\alpha_{\text{1}}+\Delta \alpha \right)},\frac{\gamma -n_{Z}\Delta \alpha \left(v_{h}\;-v_{l}\right)+n_{Z}\Delta \alpha \Delta v^{\prime }+n_{Y}(\alpha_{\text{2}}+\Delta \alpha )\Delta v^{\prime }\;}{\left(n_{Z}+n_{Y})(\alpha_{\text{2}}+\Delta \alpha \right)}\}\geq \Delta (t-v)\geq \end{array}$ $\begin{array}{c} min\{\frac{\gamma -n_{X}\Delta \alpha \left(v_{h}\;-v_{l}\right)+\alpha_{\text{1}}\Delta v^{\prime }(n_{X}+n_{Y})\;}{\left(n_{X}+n_{Y})(\alpha_{\text{1}}+\Delta \alpha \right)},\frac{\gamma -n_{Z}\Delta \alpha \left(v_{h}\;-v_{l}\right)+n_{Z}\Delta \alpha \Delta v^{\prime }+n_{Y}(\alpha_{\text{2}}+\Delta \alpha )\Delta v^{\prime }\;}{\left(n_{Z}+n_{Y})(\alpha_{\text{2}}+\Delta \alpha \right)}\} \end{array}$ or $max\{\frac{\gamma +n_{X}\alpha_{\text{1}}\Delta v^{\prime }\;}{n_{X}\alpha_{\text{1}}},\frac{\gamma \;}{n_{Z}\alpha_{\text{2}}}\}\geq \Delta (t-v)\geq min\{\frac{\gamma +n_{X}\alpha_{\text{1}}\Delta v^{\prime }\;}{n_{X}\alpha_{\text{1}}},\frac{\gamma \;}{n_{Z}\alpha_{\text{2}}}\}$.

(iv) *Both (P, R) and (R, P) are possible equilibria if and only if* $max\{\frac{\gamma -n_{X}\Delta \alpha \left(v_{h}\;-v_{l}\right)+\alpha_{\text{1}}\Delta v^{\prime }(n_{X}+n_{Y}}{\left(n_{X}+n_{Y})(\alpha_{\text{1}}+\Delta \alpha \right)},\frac{\gamma -n_{Z}\Delta \alpha \left(v_{h}\;-v_{l}\right)+n_{Z}\Delta \alpha \Delta v^{\prime }+n_{Y}(\alpha_{\text{2}}+\Delta \alpha )\Delta v^{\prime }\;}{\left(n_{Z}+n_{Y})(\alpha_{\text{2}}+\Delta \alpha \right)}\}\leq \Delta (t-v)\leq min\{\frac{\gamma +n_{X}\alpha_{\text{1}}\Delta v^{\prime }\;}{n_{X}\alpha_{\text{1}}},\frac{\gamma \;}{n_{Z}\alpha_{\text{2}}}\}$*.*

### Results

This paper observes that the (*P*, *P*) region is significantly expanded in this extended model, regardless of the information setting. Firms can now capture more value when both pursue product-oriented CSR strategies due to their different industrial positioning. Without considering business differentiation, a homogeneous product-oriented strategy would lead to greater value capture by buyers. This suggests that aligning business strategies with product-oriented CSR strategies can enhance value capture for differentiated firms. Additionally, the (*R*, *R*) region is reduced, indicating that when the connection between business and CSR strategies is considered, a homogeneous *R* strategy becomes less favorable. This reflects the idea that a differentiated strategic portfolio and dislocated competition can create diverse advantages and enable firms to capture more value.

## Conclusion and discussion

In response to the debate over the performance implications of CSR and the calls for theoretical development regarding the strategic choices among different CSR dimensions [10,29,46], this paper provides an exploration of how firms choose CSR strategies influenced by the competitive landscape and contextual factors. Specifically, it analyzes the trade-off between product-oriented CSR strategies, which focus on enhancing social responsibility, and relationship-oriented CSR strategies, which aim to build trust, using extended biform game models. The results demonstrate that market structure shapes the choice to pursue either product- or relationship-oriented CSR strategies and competitive outcomes. This paper also finds notable differences in the motivations and effects of these CSR strategies. Furthermore, it extends the baseline model to illustrate how business strategies can play a crucial coordinating role in CSR strategy decisions, impacting firms’ overall strategic portfolios.

In contrast to earlier empirical studies, which suggest that firms select CSR strategies based on their positive or negative performance implications [18], this paper’s fundamental finding is that firms choose CSR strategies based on strategic interactions in a competitive environment. Strategic equilibria are shaped by factors such as information asymmetry, product value, market segment size, transaction costs, and bargaining power. When the value derived from product- and relationship-oriented strategies is significantly high, firms tend to adopt those respective strategies. In a low information asymmetry context, firms with lower bargaining power are more inclined to choose a relationship-oriented strategy, whereas in a high information asymmetry context, these firms are more likely to opt for a product-oriented strategy. Regardless of the information asymmetry context, firms with larger market sizes tend to favor a product-oriented strategy.

Firms’ CSR strategy choices also influence overall industry value creation and the distribution of that value between firms and buyers. The total value captured by firms can increase, while buyers’ value capture may decrease if firms coordinate their strategic choices when multiple strategic equilibria exist. Additionally, the parameters of competitive settings affect the value capture for both firms and buyers. Overall, product-oriented strategies that enhance product value contribute to greater buyers’ value capture and industry value creation. When business strategies are considered, firms are more likely to adopt product-oriented CSR strategies, as these allow them to capture more value due to their distinctive market positioning.

This paper extends the theoretical boundaries of the CSR literature by exploring how market structure influences CSR strategy choices and the resulting value creation and distribution, diverging from the traditional focus on best management practices. Previous studies have emphasized the different performance implications of CSR dimensions [10,11,29]. In contrast, this study highlights the role of market competition between rival firms in shaping CSR strategies. This approach goes beyond the resource-based view, which primarily examines CSR’s impact on securing resources from specific stakeholder groups.

The explicit consideration of information asymmetry in firms’ CSR strategy choices is a notable contribution, as it remains underdeveloped in the CSR literature. Although previous research has acknowledged the importance of information disclosure and communication in shaping the formation and effectiveness of CSR strategies [49,54], this work delves deeper into how information asymmetry influences the effectiveness of different CSR dimensions and their impact on market dynamics. Information asymmetry not only affects CSR outcomes, such as consumer reactions and firm performance, but also plays a critical role in firms’ CSR strategy choices [55,56].

This paper further explores the connection between business strategies and CSR strategies. While a few studies have examined the relationship between CSR and business strategies [52,57], they have not fully uncovered the micro-foundations through which these strategies can be integrated to create and capture value for firms. The model suggests that aligning business strategies with product-oriented CSR strategies allows differentiated firms to generate and capture more value in a competitive market, thereby reducing the strategic space available for relationship-oriented CSR strategies. This study views the synergy between business and CSR strategies as a compelling topic within value-based theory.

These theoretical insights offer actionable implications for firms and policymakers in practice, particularly in varying industry and country-specific contexts. For instance, in highly competitive industries like consumer electronics where information asymmetry is often high due to rapid technological advancements, firms with weaker bargaining power, such as smaller suppliers, may prioritize product-oriented CSR (e.g., sustainable sourcing of materials) to differentiate offerings and secure market share, as seen in cases like electronics manufacturers in East Asia responding to EU regulations on environmental standards. Conversely, in relationship-intensive sectors like retail in developing countries, where low information asymmetry prevails through established buyer networks, firms might lean toward relationship-oriented CSR (e.g., community engagement programs) to build long-term trust and reduce transaction costs, aligning with policy frameworks that incentivize CSR through tax benefits for social initiatives. Policymakers can leverage these findings by tailoring regulations—such as subsidies for product-oriented CSR in large-scale markets or mandates for transparency in high-asymmetry environments—to enhance industry value creation and equitable value distribution, ultimately fostering sustainable economic growth. Creating a policy environment that recognizes and strategically reinforces the different pathways through which CSR creates value can amplify its positive impact on sustainable development.

This paper concludes by highlighting several opportunities for future theoretical and empirical research. One promising avenue for theoretical exploration is integrating network theory into the biform game model to capture variations in players’ network positions and bargaining power. Researchers could then investigate how alliances among buyers influence firms’ CSR activities within a networked market structure [38,58]. Additionally, recognizing that buyers may exhibit heterogeneous behavior when processing CSR information, an agent-based simulation model could be developed to further enhance the framework by simulating stochastic and interactive processes [59]. As the model of this paper treats different CSR dimensions as mutually exclusive strategic choices, another promising area is investigating how firms can achieve ambidexterity across CSR dimensions from a tension-centric perspective [57]. For empirical research, collecting and coding firms’ CSR strategies based on their effectiveness in value creation and capture would be valuable [13]. Moreover, exploring the antecedents of CSR strategy choices in diverse firms and the related boundary conditions would offer additional insights [24].

## Supporting information

S1 File Code python. (TXT)
